# Differential Compartmentalization of HIV-Targeting Immune Cells in Inner and Outer Foreskin Tissue

**DOI:** 10.1371/journal.pone.0085176

**Published:** 2014-01-15

**Authors:** Aiping Liu, Yu Yang, Lu Liu, Zhefeng Meng, Liangzhu Li, Chao Qiu, Jianqing Xu, Xiaoyan Zhang

**Affiliations:** Shanghai Public Health Clinical Center and Institutes of Biomedical Sciences, Key Laboratory of Medical Molecular Virology, Shanghai Medical College of Fudan University, Shanghai, China; University of Toronto, Canada

## Abstract

*Ex vivo* foreskin models have demonstrated that inner foreskin is more susceptible to HIV-1 infection than outer foreskin. In the present study we characterized the compartition of HIV-1 target cells and quantified these cells in the epidermis and dermis of inner and outer foreskins using immunohistochemistry and flow cytometry. Our data showed that the epidermis of the inner foreskin was more enriched with CD4^+^ T cells and Langerhans cells (LCs), with the co-expression of CCR5 and α4β7 receptors, than the outer foreskin. Interestingly, the vast majority of CD4^+^ T cells and LCs expressed CCR5, but not CXCR4, indicating that the inner foreskin might capture and transmit R5-tropic HIV strains more efficiently. In addition, lymphoid aggregates, composed of T cells, macrophages and dendritic cells (DCs) in the dermis, were closer to the epithelial surface in the inner foreskin than in the outer foreskin. As dendritic cells are able to capture and pass HIV particles to susceptible target cells, HIV may be able to more efficiently infect the inner foreskin by hijacking the augmented immune communication pathways in this tissue. After the inoculation of HIV-1 particles in a foreskin explant culture model, the level of p24 antigen in the supernatant from the inner foreskin was slightly higher than that from the outer foreskin, although this difference was not significant. The present study is the first to employ both CCR5 and α4β7 to identify HIV target cells in the foreskin. Our data demonstrated that the inner foreskin was more enriched with HIV target immune cells than the outer foreskin, and this tissue was structured for efficient communication among immune cells that may promote HIV transmission and replication. In addition, our data suggests the R5-tropism of HIV sexual transmission is likely shaped through the inherent receptor composition on HIV target cells in the mucosa.

## Introduction

Sexual transmission accounted for 84.9% of newly infected HIV cases in 2012 [1], and the large majority of people living with HIV in China were male (only 28.6% were women) [2]. Several factors were associated with the risk of male HIV-1 acquisition[3], such as the lack of circumcision[4], [5]. Randomized, controlled trials in Africa have shown that male circumcision reduced HIV-1 acquisition in men approximately 60% [6], [7], [8], but provided no protection to the female partners of HIV-1 positive men[9]. Meta-analysis estimated that the risk of HIV-1 transmission among non-circumcised men was at least twice that of circumcised men[10]. Although other penile sites, such as the urethra, might also play a role in HIV acquisition[11], the importance of the foreskin is shown by the observation that increased foreskin surface area is associated with an increased risk of HIV-1 infection[12]. Circumcision is now recommended as a component of HIV-1 prevention strategies. However, the mechanism through which circumcision reduces HIV-1 acquisition is not fully understood. It has been suggested that the foreskin folded over the glans on the non-erect penis, referred to as the “inner foreskin”, is particularly vulnerable to HIV. During intercourse, the foreskin is retracted and this inner aspect is exposed to potentially infectious secretions. Both this inner aspect of the foreskin and a contiguous portion exposed on both the erect and non-erect penis, termed the “outer foreskin”, are removed during circumcision. It had been hypothesized that the inner foreskin was more vulnerable to HIV due to a thinner layer of keratin compared with other penile skin. However, as recent studies have shown no significant differences in keratin thickness between the inner and outer foreskins [13], [14], the increased infection sensitivity more likely reflects intrinsic cellular characteristics, such as the number of HIV-1 target cells and their sub-cellular localization, and the expression of key molecules that mediate HIV-1 attachment and entry. The inner surface of the foreskin, which is exposed to vaginal secretions during intercourse, contains both T cells and Langerhans cells (LCs) that express HIV receptors as potential targets for viral entry [15], [16], [17].

Recently, several studies have provided controversial results on the density of potential target cells for HIV-1, including LCs, T cells, dendritic cells (DCs), and macrophages, within the inner and outer foreskins. A study of healthy men in France reported that the densities of CD3^+^ and langerin (CD207)^+^ cells in the epidermis of the inner foreskin were significantly higher than those in the outer foreskin[15]. Furthermore, using *ex vivo* foreskin explant models, other studies have shown that the inner foreskin was more susceptible to HIV-1 infection[15], [17], [18]. Additional studies have shown similar trends concerning the distribution of potential HIV-1 target cells in the foreskin tissues of North American, European and African men[15], [18], [19], [20]. However, contrasting results were reported when foreskin samples from Australian and Chinese men were examined[21], [22]. Thus, additional studies using foreskin samples from different ethnic and geographic populations are required to address this issue.

At the female genital mucosal entry point, α4β7 is able to directly bind gp120, and α4β7^+^ CD4^+^ T cells express multiple additional markers of HIV-1 susceptibility [23]. It has been shown that α4β7 closely associates with CD4 and CCR5 on CD4^+^ T cells and binds the HIV-1 envelope protein gp120 during viral attachment[24], [25], [26]. After rectal challenge, initially infected α4β7/CD4^+^ T cells migrate from the mucosa to Peyer's patches and mesenteric lymph nodes, where high-level HIV-1 replication occurs within days[27], [28], [29]. However, the expression of α4β7 and its contribution to male genital HIV-1 infection remains unknown. In the present study, we conducted a comprehensive phenotypic characterization of potential HIV-1 target cells, including enumerating T cells, macrophages, DCs and LCs and determining the localization of these cells in foreskin tissue samples obtained from Chinese men undergoing circumcision. The expression of the receptor and co-receptor of HIV-1, including CD4, CCR5, CXCR4 and α4β7, on potential HIV target cells was also investigated. These data provide insight into both the mechanism of HIV-1 transmission and the protection conferred through circumcision.

## Results

### Distribution and quantification of potential HIV-1 target cells in the foreskin

**Figure 1:**
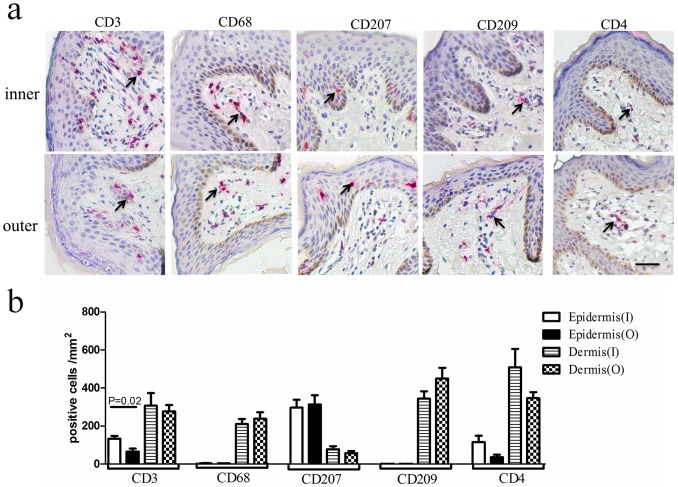
Compartmental quantification of potential human immunodeficiency virus type-1 (HIV-1) target cells in human foreskin. (**a**) Representative images for inner and outer foreskin tissues processed for immunohistochemistry staining using antibodies (Abs) against CD3, CD68, CD207, CD209 and CD4. The cells were visualized with 3-amino-9-ethylcarbazole peroxidase substrates (red). Bar = 50 µm. (**b**) The cell densities per mm^2^ of either epidermis or dermis for CD3^+^, CD68^+^, CD207^+^, CD209^+^ and CD4^+^ cells are shown as the means ± SD (*n* = 13). For each foreskin, parallel staining was performed in the inner and the outer foreskin, and the cells were counted in a minimum of 10 fields. I = Inner, O = Outer (Related-Samples Wilcoxon Signed Rank Test).

The features of potential HIV-1 target cells including their distribution and density in the foreskin were firstly investigated using immunohistochemical staining (Figure 1). The surface markers employed for immunohistochemical staining to identifiy potential HIV-1 target cells included CD3 (for T lymphocytes, T cells), CD207 (for Langerhans cells, LCs), CD209 (for Dendritic cells, DCs), CD68 (for macrophages), and the HIV-1 receptor CD4. CD3^+^ cells were enriched in the dermis (328±105 cells/mm^2^) compared with the epidermis (133±51 cells/mm^2^) (**Figure 1a**). In contrast, the majority of CD207^+^ LCs were confined to the epidermis (282±140 cells/mm^2^), and much less LCs were observed in the dermis (77±58 cells/mm^2^) (**Figures 1a and b**). Both CD209^+^ DCs and CD68^+^ macrophages were exclusively observed within the dermis; CD209^+^ DCs were primarily localized near the basal lamina (327±133 cells/mm^2^), and CD68^+^ macrophages were localized in the dermis near the stratum spinosum of epidermis (201±89 cells/mm^2^) (**Figures 1a and b**). Unlike the expression of cell lineage definition markers, the expression of CD4 was not only observed on T cells, but also on DCs, LCs and macrophages. The CD4^+^ cells were observed in both the epidermis (116±48 cells/mm^2^) and dermis (508±121 cells/mm^2^). We then further compared the densities of the different cell populations within epidermis or dermis between the inner and outer foreskin, respectively. Notably, the density of CD3^+^ cells in the epidermis was significantly lower in the outer foreskin compared with the inner foreskin (outer foreskin vs. inner foreskin: 22±18 cells/mm^2^ vs. 133±51 cells/mm^2^, *p* = 0.02; *n* = 13) (**Figures 1a and b**). There were no other significant differences found in cell population densities between the inner and outer foreskin using immunohistochemical staining.

### The inner foreskin epithelium is enriched for Langerhans cells and CD4^+^ T cells expressing HIV-1 co-receptors

**Figure 2:**
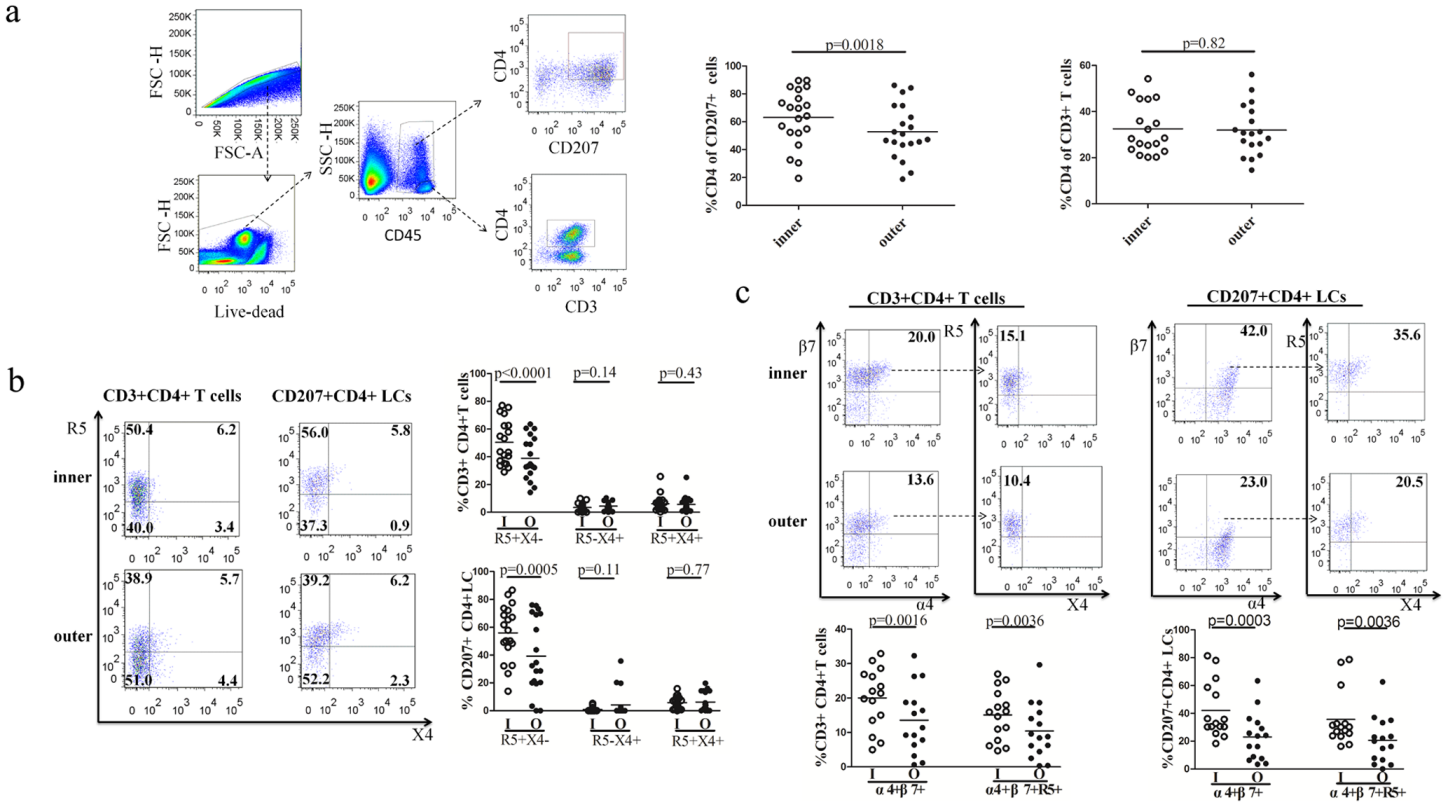
Expression of HIV-1 receptors/co-receptors on CD3^+^ T cells and CD207^+^ LCs cells in the epidermis of the foreskins. (**a**) Gating strategy on singlet, live cells, CD45^+^ cells, CD4^+^ T cells and CD4^+^ LCs. Cells isolated from fresh tissue were stained for lymphocytic markers and analyzed using flow cytometry. Relative percentages of CD3^+^/CD4^+^ cells and CD207^+^/CD4^+^ cells in CD3^+^ and CD207^+^ cells are summarized on the right. As shown, the proportions of CD207^+^/CD4^+^ in CD207^+^ cells in inner foreskins were significantly higher than in the outer foreskins. (**b**) Relative percentages of CCR5^+^CXCR4^−^, CCR5^−^CXCR4^+^, and CCR5^+^CXCR4^+^ cells in CD3/4^+^ and CD207/4^+^ cells were examined on the left and summarized on the right. The results show that proportions of CCR5^+^CXCR4^−^ cells in CD3^+^/CD4^+^ and CD207^+^/CD4^+^ cells in the inner foreskin were significantly higher than in the outer foreskin. (**c**) Relative percentages of α4β7 and α4β7/CCR5^+^ cells in CD3^+^/CD4^+^ and CD207^+^/CD4^+^ cells were examined on the left and summarized on the right. The results show that proportions of α4β7 in CD3^+^/CD4^+^ and CD207^+^/CD4^+^ cells in the inner foreskin were significantly higher than in the outer foreskin. Gating through α4β7^+^ cells of CD3^+^/CD4^+^ or CD207^+^/CD4^+^ cells in both inner and outer foreskins showed that majority of α4β7^+^ cells were also co-expressed with CCR5. The proportions of α4β7 co-expressed with CCR5 on CD3^+^/CD4^+^ and CD207^+^/CD4^+^ cells were higher in the inner foreskin than in the outer foreskin (Paired t test).

The co-expression of CD4 with CCR5 and CXCR4 on T cells and LCs was further analyzed using multi-paramater flow cytometry (Figure 2). Among all lymphocytes, CD3^+^ T cells accounted for over 90% (**Figure S1**). To minimize the discrepancies resulting from the specimen size and the physiological characteristics of different foreskin samples, we defined the proportions of CD3^+^ T cells or CD207^+^ LCs, rather than calculating the absolute counts per sample. The proportions of CD3^+^ T cells or CD207^+^ LCs that co-expressed CD4 were quantified within the epidermis of the inner and outer foreskins (*n* = 20). No significant difference in the proportion of the epidermal CD3^+^/CD4^+^ T cells between the inner and outer foreskins (32.4% vs. 30.8%, *p* = 0.82) was observed (**Figure 2a**). Interestingly, the proportion of CD4-expressing CD207^+^ LCs was significantly higher in the epidermis of the inner foreskin than that in outer foreskin (Mean: 63.1% vs. 52.8%, *p* = 0.0018) (**Figure 2a**). The expression of HIV-1 co-receptors CCR5 and CXCR4 on CD4^+^ T cells and CD207^+^/CD4^+^ LCs was further investigated. We observed that the co-expression of CCR5 but not CXCR4 was significantly higher in the inner foreskin than that in the outer foreskin (50.4% vs. 38.9% on CD4^+^T cells, *p*<0.0001; 56.0% vs. 39.2% on CD207^+^/CD4^+^ LCs, *p* = 0.0005, respectively) (**Figure 2b**). The results in Figure 1 and Figure 2 are not directly comparable (i.e. a higher proportion of CD207^+^ cells were found to be CD4^+^ than would be expected based on immunohistochemical staining results), this is likely due to the increased sensitivity of flow cytometry, detecting low levels of CD4 expression that were not detected by immunohistochemistry staining. The comparisons between the inner and outer foreskin were made in the same assay, thereby avoiding discrepancies resulting from the inherent difference in the sensitivities of these assays.

The co-expression of α4β7 with CCR5 on CD4^+^ T cells was investigated. As shown in **Figure 2c**, the expression of α4β7 on CD3/4^+^ T cells in the inner foreskin was almost two-fold higher than that in the outer foreskin (20.0% vs. 13.6%, *p* = 0.0016). In addition, the co-expression of CCR5 with α4β7 on CD4^+^ T cells was also significantly higher in the inner foreskin than that in the outer foreskin (15.1% vs. 10.4%, *p* = 0.0036). Similar trends were observed for CD207^+^/CD4^+^ LCs. The expression of α4β7 on CD207^+^/CD4^+^ LCs was nearly two-fold higher in the inner foreskin than in the outer foreskin (42.0% vs. 23.0%, *p* = 0.0003). The co-expression of α4β7 with CCR5 on CD207^+^/CD4^+^ LCs was also significantly higher in the inner foreskin than that in the outer foreskin (35.6% vs. 20.0%, *p* = 0.0036). These data indicated that the inner foreskin might be more susceptible to R5 tropic HIV-1 infection.

The memory phenotype of CD4^+^ T cells in the epithelium was assessed after staining with CD45RA and CCR7 to distinguish naive (CD45RA^+^CCR7^+^), central memory (T_CM_, CD45RA^−^/CCR7^+^) and effector memory cells (T_EM_, CD45RA^−^/CCR7^−^). The results showed that the T_EM_ phenotype was predominantly observed (>90% of CD4^+^ T cells) in the epithelium of both the inner and outer foreskins, whereas only a few naïve CD4^+^ T cells were identified (ranging from 0 to 1.2%) (**Figure S2a**). The early activation marker CD69 on CD4^+^ T cells was also quantified because CD69^+^CD4^+^ T cells have been demonstrated as a major early target for HIV-1 infection [30], [31]. The co-expression of CD69 on CD4^+^ T cells from the inner foreskin was 62.6%±16.9%, which was slightly higher than that in the outer foreskin (51.6%±20.6%) (**Figure S2b**). Overall, the CD4^+^ T cell memory phenotypes and activation status were comparable between the inner and outer foreskin epithelium.

### The inner foreskin dermis is enriched for macrophages and CD4^+^ T cells expressing HIV-1 co-receptors

**Figure 3:**
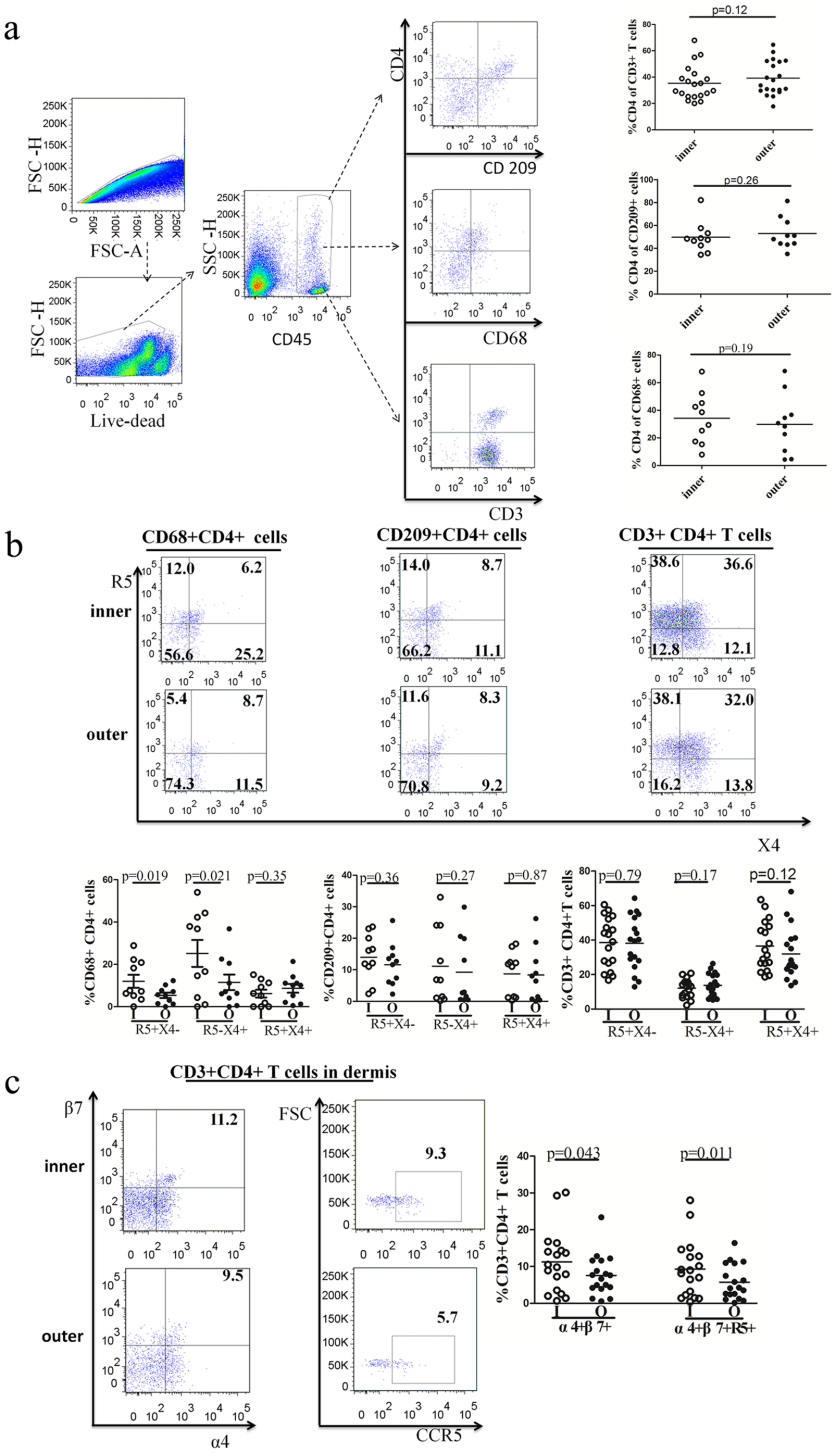
Expression of HIV-1 receptors/co-receptors on CD3^+^ cells, CD68^+^ cells and CD209^+^ cells in the dermis of the foreskins. (a) Gating strategy on singlet, live cells, CD45^+^ cells, CD3^+^/CD4^+^ T cells, CD68^+^/CD4^+^ cells and CD209^+^/CD4^+^ cells. The relative percentages of CD3^+^/CD4^+^, CD68^+^/CD4^+^ and CD209^+^/CD4^+^ cells on CD3^+^, CD68^+^ and CD209^+^ cells are summarized on the right, showing that no significant differences were observed in all three populations between the inner and the outer foreskins. (b) The relative percentages of CCR5^+^CXCR4^−^, CCR5^−^CXCR4^+^, and CCR5^+^CXCR4^+^ cells in CD3^+^/CD4^+^, CD68^+^/CD4^+^ and CD209^+^/CD4^+^ cells are examined on the left and summarized on the right. The results show that proportions of CCR5^+^CXCR4^−^ and CCR5^−^CXCR4^+^ cells in CD68^+^/CD4^+^ cells in the inner foreskin dermis were significantly higher than in the outer foreskin dermis. (c) The relative percentages of α4β7 and α4β7/CCR5^+^ cells in CD3^+^/CD4^+^ cells in the foreskin dermis are examined on the left and summarized on the right. The results show that proportions of α4β7 on CD3^+^/CD4^+^ cells were significantly higher in the inner foreskins dermis than in the outer foreskins dermis. Gating through α4β7^+^ cells of CD3^+^/CD4^+^ cells from the dermis of both the inner and the outer foreskins showed that the majority of α4β7^+^ cells were also co-expressed with CCR5. Proportions of α4β7 co-expressing with CCR5 were significantly higher in the inner foreskin dermis than in the outer foreskin dermis (Paired t test).

An abundance of potential HIV-1 target cells, including T cells, CD209^+^ DCs and CD68^+^ macrophages, were observed in the dermis of human foreskin tissues (*n* = 20). As shown in **Figure 3a**, there were no significant differences observed in the proportions of CD4^+^ T cells (34.6% vs. 37.7%, *p* = 0.12), CD209^+^ DCs (48.9% vs. 51.1%, *p* = 0.26) or CD68^+^ macrophages (34.2% vs. 29.8%, *p* = 0.19) between the inner and outer foreskins. However, the proportions of CD68^+^/CD4^+^ macrophages that are either CCR5^+^CXCR4^−^ or CCR5^−^CXCR4^+^ were higher in the inner foreskin than in the outer foreskin (12.0% vs. 5.4%, *p* = 0.019; 25.2% vs. 11.5%, *p* = 0.021). This difference was not observed for CD4^+^ T cells or CD209^+^/CD4^+^ DCs (**Figure 3b**). These results implied that CD68^+^ macrophages in the dermis of the foreskin might support either R5 or X4 tropic HIV-1 infections. In addition, the proportion of CD4^+^ T cells in the dermis co-expressing α4β7 (11.2% vs. 9.5%, *p* = 0.043) or α4β7/CCR5 (9.3% vs. 5.7%, *p* = 0.011) was higher in the inner foreskin than in the outer foreskin (**Figure 3c**). Similarly, CD4^+^ T cell memory phenotypes and their activation status were also determined, and no significant differences were observed in the dermis between the inner and outer foreskins (**Figure S2**).

In both inner and outer foreskin, the parameters for CD4^+^ T cell activation and memory status between the epidermis and dermis were neither different except T_CM_, which was significant higher in dermis than that in epidermis(data not shown).

### Lymphoid aggregates were more concentrated in the inner foreskin than in the outer foreskin

**Figure 4:**
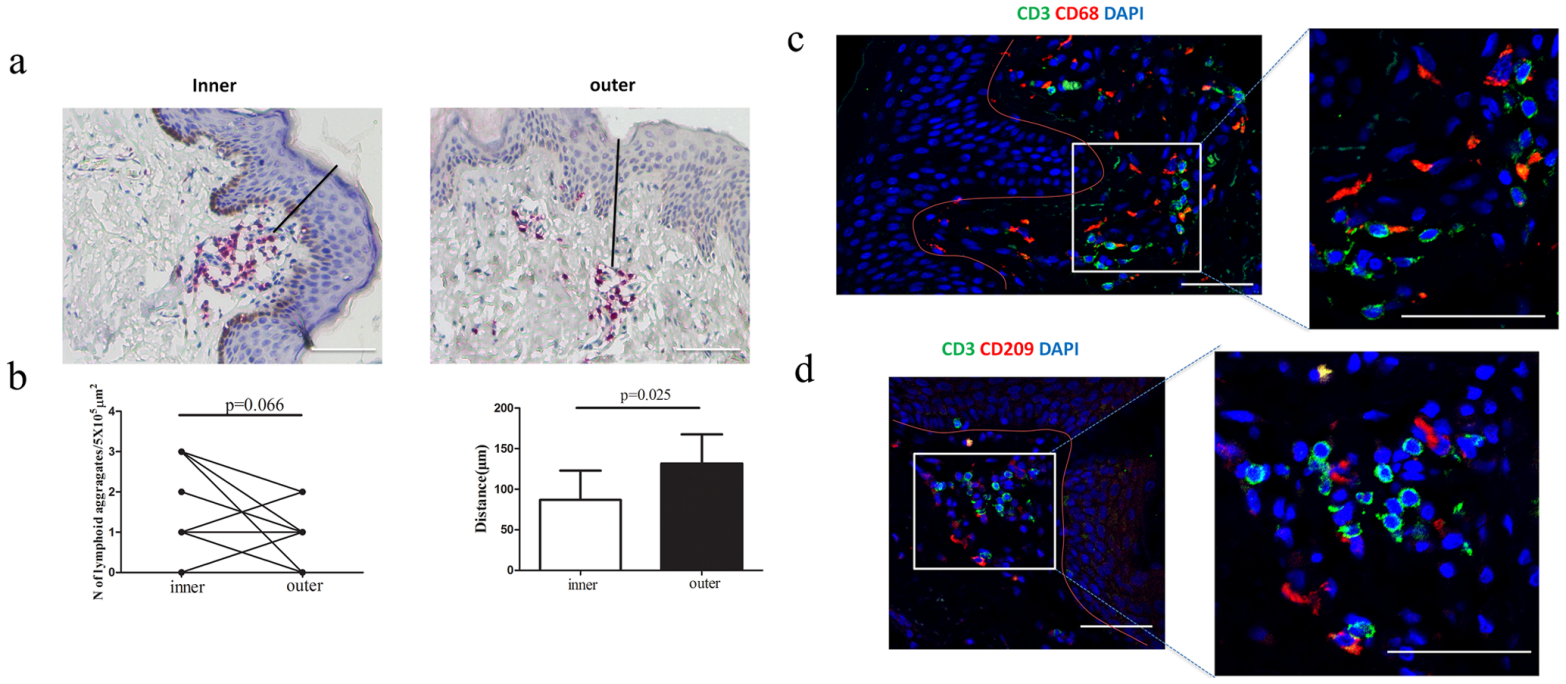
Lymphoid aggregates and the co-localization of CD3^+^ cells and CD68^+^ cells or CD209^+^ cells in the inner and the outer foreskins. (a) CD3^+^ cells aggregated in dermis in the inner and outer foreskins, Bars = 100 µm. (b) The number of lymphoid aggregates per 5×10^5^ µm^2^ and the distance from the lymphoid aggregates to the epithelial surface between the inner and the outer foreskins were compared, and significant differences were observed for the latter. Co-localization of CD3^+^ T cells with CD68^+^ macrophages (c), or CD209^+^ DCs (d) within the lymphocytes aggregates. Bar = 50 µm (Related-Samples Wilcoxon Signed Rank Test).

Lymphoid aggregates (one aggregate consisted of more than 20 T lymphocytes per 5×10^5^ µm^2^) [32] were observed in the foreskin tissues. The aggregates comprised T cells, CD209^+^ DCs and CD68^+^ macrophages (**Figure 4**). The co-localization of T cells with macrophages and DCs might facilitate HIV-1 transmission among these cells in the foreskin. The number of lymphoid aggregates per 5×10^5^ µm^2^ and their distance from the epithelial surface was measured (**Figure 4a**). The number of lymphoid aggregates in the inner foreskin ranged from 0 to 3 with a mean of 2. This number was slightly higher than that in the outer foreskin (ranging from 0 to 2, with a mean of 1) (*p* = 0.066) (**Figure 4b** **, left**). Interestingly, the mean distance between the lymphoid aggregates and the epithelial surface was 86.7 µm (ranging from 22.6 µm to 135.3 µm) in the inner foreskin and 131.8 µm in the outer foreskin (ranging from 89.0 µm to 179.0 µm) (*p* = 0.025) (**Figure 4b** **, right**), indicating that the lymphoid aggregates in the inner foreskin were more readily accessible to HIV-1 than their counterparts in the outer foreskin, suggesting a role for these cells during early HIV-1 infection in foreskin tissues.

### Replication of HIV-1 in foreskin explants

Foreskin tissue samples (*n* = 3) were acquired from adolescent and adult males after elective or corrective surgery. The inner and outer foreskin explants cultures were then established, separately. As observed in the present study, the vast majority of CD4^+^T cells and LCs expressed CCR5, but not CXCR4; two R5 viruses (NLENG1-YU-2-IRES (NIR) with subtype B envelope[33], and SH6.81 with CRF-01AE envelope [34]) were selected for these assays. The two R5 viruses also represented the most prevalent sexually transmitted HIV-1 strains in China. The pseudovirus VSVG-HIV, derived from pNL4-3, pseudotyped with Vesicular Stomatitis Virus-G envelope were used as positive control and heat inactivated with NIR (iNIR) were used as negative controls. The experiment was conducted in duplicates, and the representative results are shown as the means ± SD.

**Figure 5:**
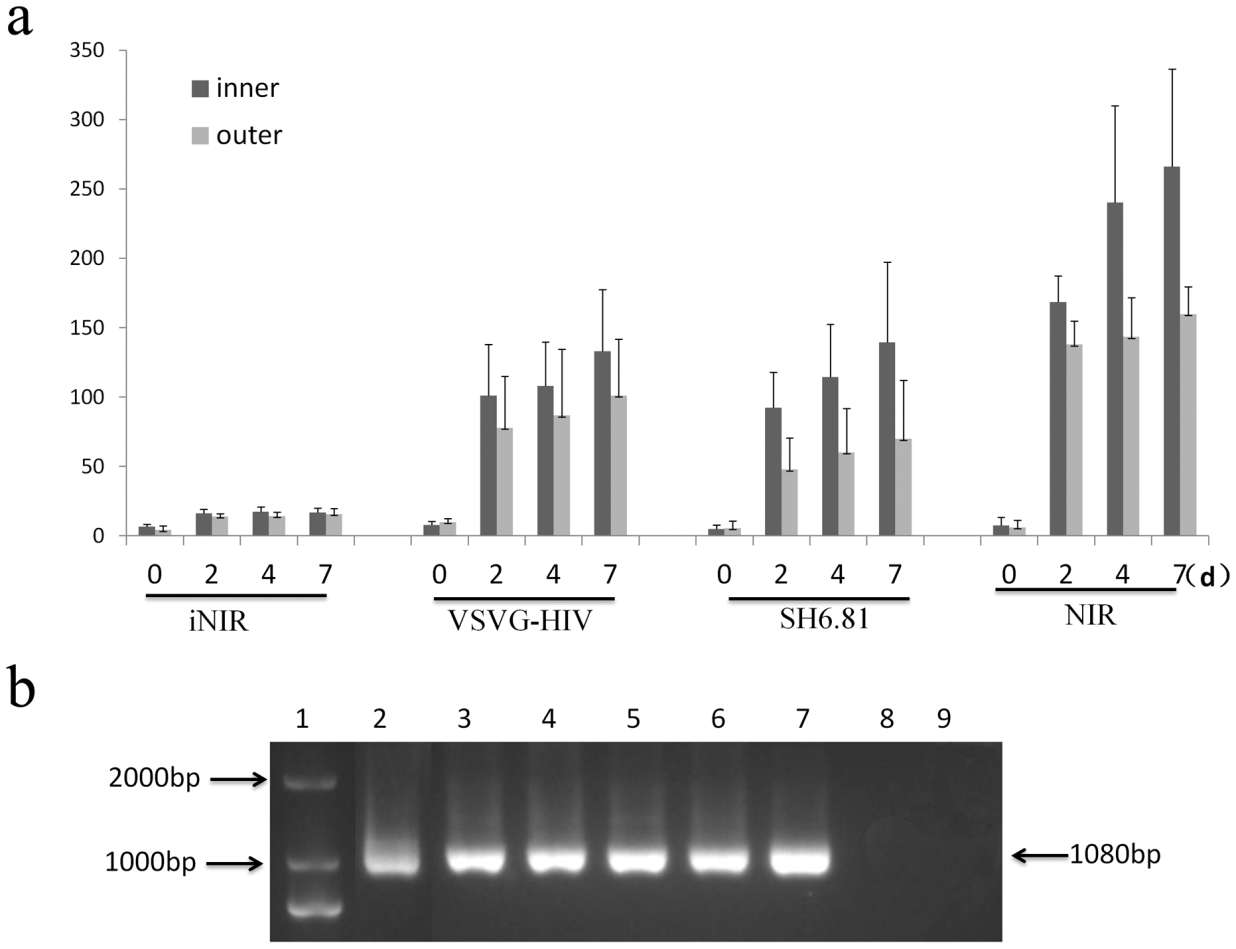
HIV replication in explants derived from human male foreskins. (**a**) p24 antigen levels in culture fluid of the inner and outer foreskin-derived explants exposed to HIV-1 strain NLENG1-YU2-IRES (NIR), heat inactivated NLENG1-IRES (iNIR), and pseudovirus SH6.81 and pseudovirus VSV-G-HIV (means ± SD were present). (**b**) Gag DNA nested PCR results. Lanes 1–9 represented DL2000 (lane 1), inner and outer foreskins infected with NIR (lanes 2–3), pseudovirus SH6.81 (lanes 4–5), pseudovirus VSV-G-HIV (lanes 6–7) and iNIR (lanes 8–9) (Related-Samples Wilcoxon Signed Rank Test).

The levels of p24 in the supernatant of explant cultures was determined on days 0, 2, 4 and 7 after exposure to the viruses (**Figure 5a**). For all viral strains used, no significant increase of p24 antigens was observed in the culture supernatant of foreskin explants treated with the iNIR or VSVG-HIV (*p*>*0.05*), during the incubation period. In contrast, an increase in the levels of p24 antigens was observed in the culture supernatant of foreskin-derived explants at 2 to 7 days post-exposure to both live virus strains NIR and SH6.81. The levels of p24 were higher in the supernatant of inner foreskin-derived explants compared with outer foreskin-derived explants (**Figure 5a**). These differences were not statistically significant (Related-Samples Wilcoxon Signed Rank Test; both *p* = 0.125). After foreskin explant exposure to viral infection for 4 days, the explants were assayed for viral genomes using nested PCR. An integrated HIV genome was detected in all foreskin explants, except those inoculated with iNIR (**Figure 5b**).

## Discussion

It has been hypothesized that keratin provides an impermeable barrier to HIV-1 and the inner foreskin is more thinly keratinized; thus the removal of the inner foreskin through circumcision is protective against HIV-1. However, studies have reported conflicting results associated with keratin thickness in the inner and outer foreskins[13], [21], [22], [35]. These conflicting reports might reflect differences in the foreskin samples obtained from different geographical areas, such as North America[13], Australia[21], China[22], and France[37]. In the present study, no significant difference was observed in keratin thickness between the inner and outer foreskin samples obtained from Chinese men (data not shown),which is different from what reported from another Chinese study in which more than half of participants (14 out of 20) experienced desquamation in their outer foreskin [22]. As we did not observe significant desquamation in our study subjects, this may explain the observed discrepancies between this study and previous one.

Another biological mechanism that might explain the protective effect of circumcision against HIV-1 is the difference in the HIV-1 target cells between the inner and outer foreskins. Previous reports on the densities of HIV target cells in the inner and outer foreskin tissues are also contradictory[15], [18], [19], [20], [21], [22], [32], [36]. Studies using foreskin samples from North American, European and African men have shown that HIV target cells are more concentrated in the inner foreskin[15], [18], [19], [20], whereas studies using foreskin samples from Australian and Chinese men have shown the opposite results[21], [22]. While it is possible that the discrepancies in keratin thickness and target cell densities between different studies reflect methodological issues, such as the desquamation of keratin using histological procedures, the use of different surface markers to identify target cells, and a lack of delineation between epithelial and dermal compartments, it is also possible that these discrepancies reflect genuine differences in foreskin immunology among geographical regions. The composition of immune cells in the inner foreskin is likely adapted to the external environment, and thus shaped through the pathogen exposure history. Therefore, subjects from different regional areas of the world or different ethnic groups could be highly different[15], [18], [19], [20], [21], [22].

In this study, we performed immunohistochemical staining of the inner and outer foreskins using HIV target cell-specific markers, namely, langerin (CD207) for LCs, CD3 for T cells, DC-SIGN (CD209) for DCs and CD68 for macrophages. In addition, we further determined the co-expression of the HIV receptor CD4 with co-receptors, including CCR5, CXCR4 and α4β7. The respective cell proportions were evaluated in either the epidermis or dermis using flow cytometry. Our data revealed several immunological features that might render the inner foreskin more susceptible to HIV infection. First, we observed that the epithelial layer of the inner foreskin was more enriched with CD3^+^ T cells than the outer foreskin. The majority of CD3^+^ T cells were effector memory T cells; as the foreskin is prone to infection with external pathogens[15], [17], [18], [37], activated memory T cells might preferentially migrate to the foreskin to eradicate localized pathogens. In addition, a greater proportion of epithelial CD4^+^T cells and LCs expressed the HIV-1 co-receptor CCR5 and α4β7 in the inner foreskin compared with the outer foreskin, suggesting that the process of HIV-1 capture by LCs and transfer to CD4^+^ T cells might be more efficient in the inner, compared with the outer foreskin[15], [17]. Interestingly, the vast majority of foreskin CD4^+^ T cells and LCs in the epidermis expressed CCR5, but not CXCR4, indicating that the inner foreskin might be more efficiently infected with the R5-tropic strain of HIV, which might explain why the R5 strain is consistently observed as the founder virus during sexual transmission [38]. This preferred R5-tropism might be further reflected through the observation that α4β7 was primarily co-expressed with CCR5, but not with CXCR4, on CD4^+^ T cells.

In addition to having a higher density of CD4^+^ T cells and LCs expressing HIV-1 co-receptors, we also observed that lymphoid aggregates in inner foreskins are closer to the apical surface compared to outer foreskins. Lymphoid aggregates (and likely lymphoid structures), including T cells, macrophages, and other immune cells, have previously been observed in both the foreskin and the female genital tract [32], [39]. The co-location of T cells with macrophages or DCs in these aggregations suggests that their biological organization facilitates efficient communication between the innate and adaptive arms of the immune system, providing sites for the rapid local expansion of immune effector cells to fight invading pathogens.

Overall, our data demonstrated that the inner foreskin is more enriched with HIV-susceptible immune cells compared with the outer foreskin. This result suggests that the HIV-1 capture and transfer to available target cells would be more efficient in the inner foreskin and provides a biological explanation for the efficacy of male circumcision in preventing HIV infection.

## Methods

### Tissue source

The foreskin tissue samples were obtained from healthy youth and adults (mean age 27 years old, ranging 10–53 years) undergoing circumcision due to phimosis and redundant prepuce at local hospitals. A brief questionnaire was administered to study subjects or their parents to obtain additional information on sexual history and history of sexually transmitted infections or urinary tract infection (UTI). A routine clinical examination, including an STI screening test, was conducted. Only people without detectable abnormal manifestation and STIs were qualified for the circumcision. The protocol was approved through the ethic committee at Shanghai Public Health Clinical Center, and written informed consents was obtained from all adult study participants; the parents provided written informed consent for the 10-year-old child. Although not required, minors were recruited into our study. All tissues were obtained according to institutional guidelines. The donor tissue samples were processed in the laboratory on the same day of the surgery and maintained in sterile saline solution within 20 minutes of surgery until further processing. Each specimen was determined for the inner and outer aspects of the foreskin using the following criteria: darker pigmentation for outer foreskin compared with inner foreskin, smoother surface for the inner foreskin, and smaller surface area for the inner foreskin. The two sides of the inner and outer foreskins were separated using a scalpel. The specimens without clear inner and outer aspects were discarded.

### Isolation of primary foreskin cells and flow cytometric assay

The foreskin sample was placed epidermal side down, and any remaining fat and muscle tissue was removed from the dermal side. The remaining layer contained the epidermis and dermis. Half of this layer was cut into 5×5 mm^2^ pieces and incubated with the epidermal side up in 60-mm culture dishes containing 5 ml DMEM medium (Hyclone, USA) supplemented with 1.2 U ml^−1^ Dispase II (Roche Diagnostics GmbH, Mannheim, Germany) overnight at 4°C. Epidermal sheets mechanically peeled from the dermis, were cut into 1-mm^2^ pieces [40], [41]. The epidermal sheets were incubated in 1 ml of 0.25% Trypsin/EDTA (Gibco) for 5 min at 37°C. The dermis was acquired from the other half of this layer using bend scissors. The remaining dermis tissues were promptly further sectioned to create pieces of approximately 0.25 mm^2^. Each piece was placed in a 1.5-ml conical tube containing 1.0 ml of 100 U ml^−1^ Collagenase Type II (Gibco, USA) and 50 U ml^−1^ of DNAse I (Invitrogen, USA) in DMEM media supplemented with 10 U ml^−1^ penicillin and 10 µg ml^−1^ streptomycin. The two digestion products were filtered through a 100-µm-cell strainer (BD Biosciences, Franklin Lakes, NJ) to remove any remaining undigested tissue [42]. The filtered cells were washed twice, resuspended in 100 µl of 2% NCS PBS. The foreskin epidermis and dermis single-cell suspensions were added to Eppendorf tubes. Before staining, the human FC receptor-binding inhibitor (eBioscience) was added to the sample in an Eppendorf tube and incubated at 4°C for 30 minutes. A reagent containing CD45 (HI30), CD3 (UCHT1), CD4 (OKT4), CD207 (DCGM4), CCR5 (2D7), CXCR4 (12G5), α4 (9F10), β7 (473207), CD45RA (HI100), CCR7 (TG8/CCR7), CD68 (Y1/82A), CD209 (9E9A8) was added separately to the Eppendorf tubes. The cells were acquired using the FACSAria system (BD Bioscience, San Jose, CA), and the data were analyzed using FlowJo 7.6.5 software (Tree Star, Ashland, OR).

### Histological analysis and Immunohistochemistry

After fixation, the foreskin tissue was embedded in paraffin, and serial 4-µm sections were prepared. The sections were deparaffinized in xylene and graded alcohol solutions. For immunohistochemistry, the samples were microwaved in 10 mM citrate buffer (pH = 6.0) for antigen retrieval, cooled in the same buffer, and washed in PBS. Selected anti-human monoclonal antibodies were used to detect the immune markers of interest: rabbit polyclonal anti-CD3 (Abcam, Cambridge, UK), mouse monoclonal anti-langerin (clone: 12D6, Abcam, Cambridge, UK), monoclonal anti-human DC-SIGN (CD209) antibody (clone: 120507, R&D, USA), monoclonal mouse anti-human CD68 (clone: PG-M1, DAKOCytomation, Denmark), and the EnVision™ G|2 Doublestain System, Rabbit/Mouse (DAB+/Permanent Red) (DAKOCytomation, Denmark). The sections were counterstained for 30 seconds with hematoxylin solution (DAKOCytomation, Denmark) and mounted using environmentally friendly sealed medium. For each foreskin tissue sample, parallel staining was performed on the inner and the outer foreskin. The images were captured using the 20× objective of an Olympus CKX41 inverted microscope (Japan) equipped with a CCD camera. For the quantification, Image J software (NIH, USA) was used to count the number of positively stained cells (N) in a minimum of 10 separate fields under the microscope. The results are expressed as the N per cumulated surface area (mm^2^) of 10 separate fields.

### Confocal microscopy

For immunofluorescence and confocal microscopy, the frozen sections were blocked in PBS containing 2% BSA (Vector Laboratories, Burlingame, CA) for 20 minutes at room temperature. After several washes in PBS, the sections were incubated overnight at 4°C with primary Abs (50 µl per section, diluted in PBS/2% BSA), including mouse monoclonal anti-langerin (clone: 12D6, Abcam, Cambridge, UK), mouse monoclonal anti-human DC-SIGN (CD209) (clone: 120507, R&D, USA), monoclonal mouse anti-human CD68 (clone: PG-M1, DAKOCytomation, Denmark), and rabbit polyclonal anti-CD3 (Abcam, Cambridge, UK). The sections were washed with PBS and incubated for 1 hour at room temperature with the appropriate secondary Abs (50 µl per section, diluted 1∶100 to 1∶200 in PBS containing 2% BSA), including the Alexa Fluor® 488 F(ab′)2 fragment of goat anti-rabbit IgG (H+L) (Invitrogen, California, USA) and Alexa Fluor® 594 goat anti-mouse IgG (H+L) (Invitrogen, California, USA). The cell nuclei were stained with DAPI (4, 6-diamidino-2-phenylindole; Sigma, USA) for 15 min at room temperature, and the sections were washed again in PBS and mounted with ProLong® Gold and SlowFade® Gold Antifade Reagents (Invitrogen, California, USA). For confocal microscopy, the staining was visualized using a Zeiss 710 microscope (Carl Zeiss AG, Jena, Germany), and the acquired image stacks were analyzed using Image J software (NIH).

### Virus preparation

NLENG1-YU-2-IRES infectious clone plasmid was an in-kind gift from Dr David Levy (New York University, College of Dentistry)[33]. The plasmid was transfected into exponentially dividing 293T cells using Lipofectamine 2000 transfection reagent (Invitrogen, California, USA). Pseudovirus SH6.81 and pseudovirus VSV-G-HIV were generated after co-transfecting exponentially dividing 293T cells with an Env-expressing plasmid (SH6.81\VSVG-HIV [34]) and an Env-deficient HIV-1 backbone vector (pSG3Denv) using Lipofectamine 2000 transfection reagent. The virus-containing supernatants were harvested at 2 days after transfection, centrifuged, passed through a 0.45-µm filter, aliquoted and stored at −80°C. The 50% tissue culture infectious dose (TCID50) of each batch of pseudovirus was determined in HIV permissive TZM-BL cells (from AIDS Research and Reference Reagent Program, NIH, USA). The HIV virions were inactivated for 30 minutes at 75°C as a background control [43].

### HIV infection of human male foreskins

The foreskin samples (*n* = 3) were placed epidermal side down, and any remaining fat and muscle tissue was carefully removed from the dermal side. Round pieces of foreskin tissues, including the intact epidermis and dermis of either the inner or outer foreskin, were gently cut using an 8-mm Harris Uni-Core, and placed epidermal side up in 24-well plate containing 1 ml DMEM supplemented with 10% FBS, 10 U ml^−1^ penicillin and 10 µg ml^−1^ streptomycin. The explants were incubated with VSV-G-HIV at 10^4^ TCID50 for 4 hours (strains NLENG1-YU-2-IRES (NIR), heat inactive NLENG1-YU-2-IRES (iNIR), pseudovirus SH6.81 and pseudovirus VSVG-HIV). After viral incubation, the explants were washed five times in fresh medium and reestablished into organ culture. After 2, 4 and 7 days, the organ culture fluid (OCF) was collected for the p24 antigen assay. The explants were dissociated, and the genome DNA was extracted for nested PCR.

### Statistical analysis

The measurements from several images of each treatment were used to calculate an average value for a specific treatment for each assay. The data were justified using the Shapiro-Wilk's test. The statistical significance analysis was performed using an SPSS program and paired t test if the data belonged to a Gaussian distribution; otherwise, a non-parametric test (two related sample Wilcoxon signed rank test) was adopted. The *p* values <0.05 were considered as positive results.

## Supporting Information

Figure S1 CD3^+^ T cells were the main lymphocytes observed in the foreskins. Gating for CD3^+^ T cells in the epidermis and dermis of the inner and outer foreskins is shown on the left, and the percentages in CD45^+^ lymphocytes are shown on the right. The percentage of CD3^+^ T cells in the epidermis was significantly higher than in the dermis.
(TIF)

Figure S2 The phenotypes of CD4^+^ T cells and their activation status in the inner and outer foreskins. (**a**) CD4^+^ T cells were phenotyped through CD45RA and CCR7. The majority of CD4^+^T cells were effect memory cells, followed by terminal effect memory cells in the epidermis and central memory cells in the dermis. (**b**) The activation of CD4^+^ T cells was determined through CD69. No difference was observed between the inner and outer foreskins.
(TIF)
