# Supplementary figures and images for: Differential Compartmentalization of HIV-Targeting Immune Cells in Inner and Outer Foreskin Tissue
Source: PLoS One. 2014 Jan 15;9(1):e85176. doi: 10.1371/journal.pone.0085176 (PMC3893184; doi:10.1371/journal.pone.0085176)

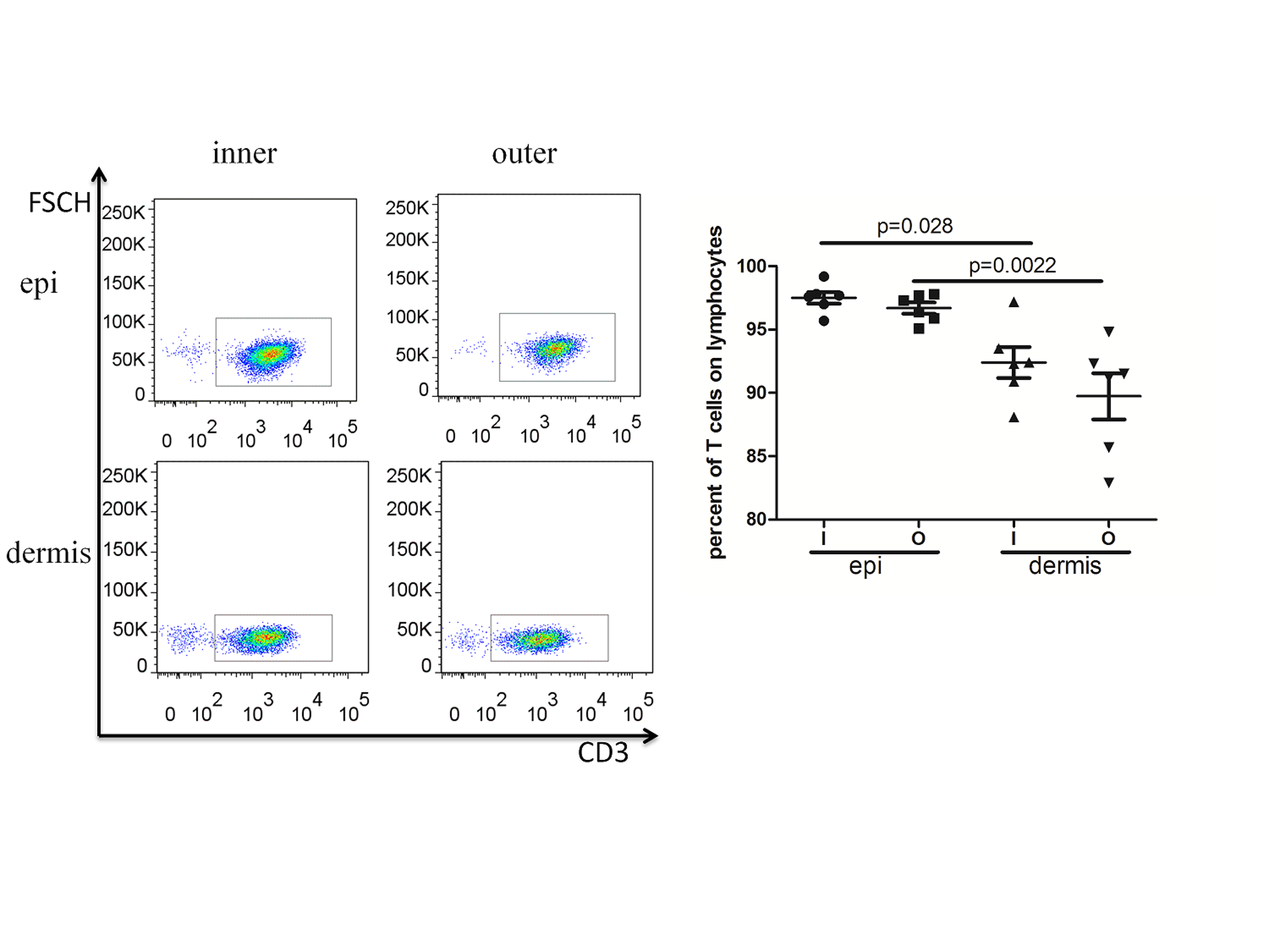

Supplement: Figure S1 — CD3+ T cells were the main lymphocytes observed in the foreskins. Gating for CD3+ T cells in the epidermis and dermis of the inner and outer foreskins is shown on the left, and the percentages in CD45+ lymphocytes are shown on the right. The percentage of CD3+ T cells in the epidermis was significantly higher than in the dermis. (TIF) [file pone.0085176.s001.tif]

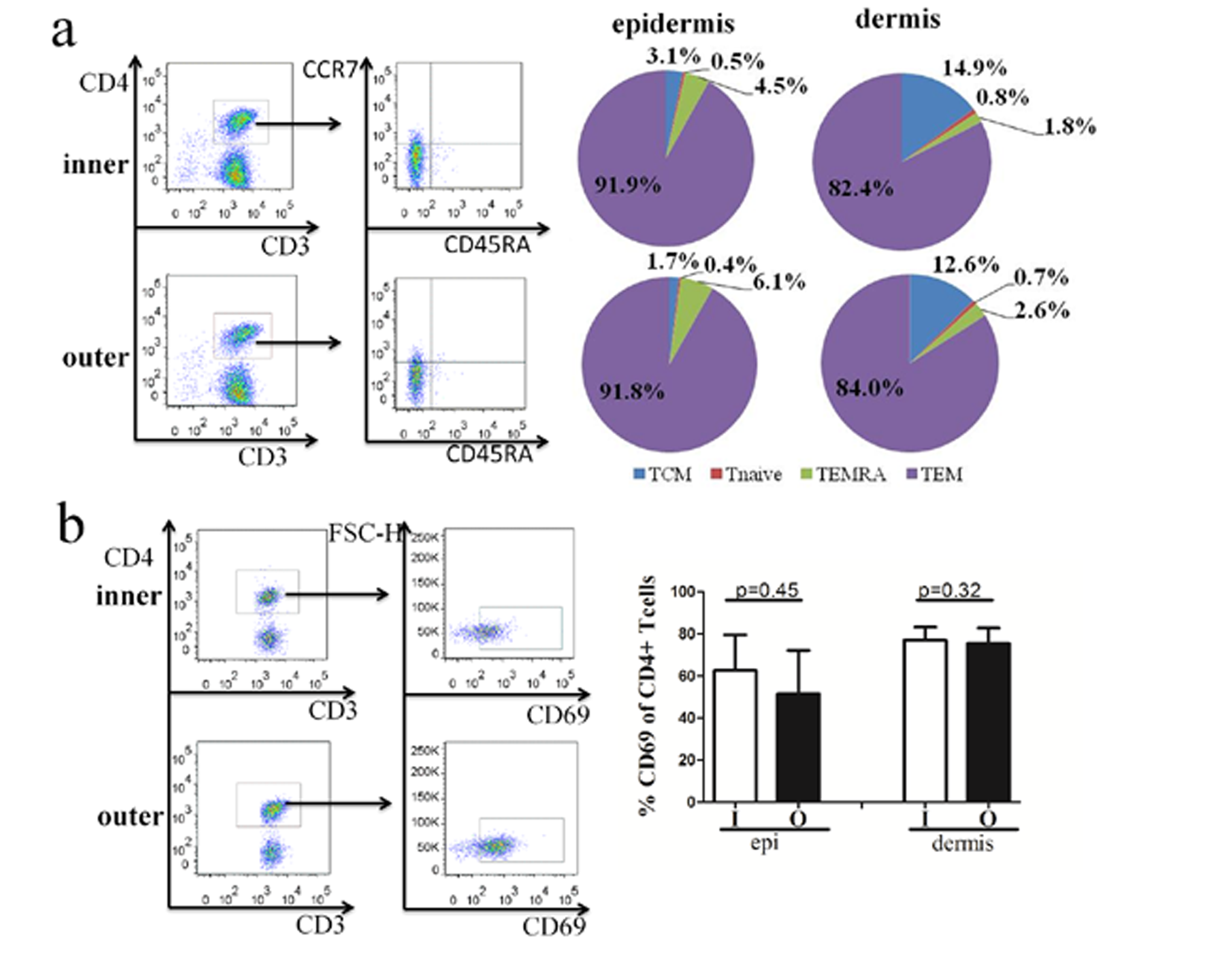

Supplement: Figure S2 — The phenotypes of CD4+ T cells and their activation status in the inner and outer foreskins. (a) CD4+ T cells were phenotyped through CD45RA and CCR7. The majority of CD4+T cells were effect memory cells, followed by terminal effect memory cells in the epidermis and central memory cells in the dermis. (b) The activation of CD4+ T cells was determined through CD69. No difference was observed between the inner and outer foreskins. (TIF) [file pone.0085176.s002.tif]
